# MYC associated zinc finger protein promotes the invasion and metastasis of hepatocellular carcinoma by inducing epithelial mesenchymal transition

**DOI:** 10.18632/oncotarget.13416

**Published:** 2016-11-16

**Authors:** Wei Luo, Xiaonian Zhu, Wei Liu, Yuan Ren, Chunhua Bei, Linyuan Qin, Xueyan Miao, Fen Tang, Guifang Tang, Shengkui Tan

**Affiliations:** ^1^ School of Public Health, Guilin Medical University, Guilin 541004, Guangxi, People's Republic of China; ^2^ Department of Hepatology, The Affiliated Nanxishan Hospital of Guilin Medical University, Guilin 541004, Guangxi, People's Republic of China

**Keywords:** MYC associated zinc finger protein (MAZ), hepatocellular carcinoma (HCC), zinc finger E-box binding homeobox 1 (ZEB1), zinc finger E-box binding homeobox 2 (ZEB2), epithelial-mesenchymal transition (EMT)

## Abstract

MYC associated zinc finger protein (MAZ) plays a key role in regulation of gene expression and tumor development. Studies have shown that deregulated expression of MAZ is closely related to the progression of tumors such as glioblastoma, breast cancer, prostate cancer and liposarcoma. However, the role of MAZ in hepatocellular carcinoma (HCC) has not been fully elucidated. Here, we found that expression of MAZ was increased in HCC and correlated to the distant metastasis of HCC. Moreover, we found that MAZ had a relationship with zinc finger E-box binding homeobox 1 and 2 (ZEB1 and ZEB2), two important mesenchymal markers in epithelial-mesenchymal transition (EMT) that were over-expressed in HCC. After knocking-down MAZ expression in HCC cell lines using RNA interruption, HCC cell proliferation, tumorigenesis, invasion and migration were significantly inhibited. In addition, we found that expression of other EMT markers was also changed besides ZEB1 and ZEB2 by decreasing MAZ expression, both detected *in vivo* and *in vitro* assays. Therefore, we conclude that MAZ can promote the invasion and metastasis of HCC by inducing EMT.

## INTRODUCTION

Hepatocellular carcinoma (HCC) is a common malignant tumor of digestive system, a consequence from the interaction between environmental and genetic factors, and its incidence has close relationship with gender, geographic location and family history of patients [1]. Recent reports show there are about 800,000 new cases of HCC around the world each year, of which more than 50% occurred in China [2, 3]. Guangxi has a high incidence of HCC, and the high mortality rate of HCC in Guangxi makes HCC to be the first death cause of tumors, which accounts for 40% of mortality caused by all the malignant tumors [1]. Most patients of HCC are diagnosed at the advanced stage with poor prognosis when they have related symptoms [4, 5]. Though the comprehensive treatment with surgical resection and liver transplantation greatly improves the clinical effect of HCC, the curative rate and long-term survival rate is still low. There are 60%–70% or more of HCC patients have recurrence during 5 years after surgery in China. In recent years, extensive research has been attracted to identify the mechanism of pathogenesis and carcinogenesis, markers or targets of diagnosis, treatment and prognosis on HCC [6–8]. However, the exact molecular pathogenesis of HCC is still not fully understood and the current prognostic markers are still not satisfactory either in terms of accuracy or repeatability. Therefore, we need to find an effective biomarker for early diagnosis and treatment to the prevention and treatment of HCC.

Zinc finger protein was first found in transcription factor IIIA (TFIIIA) of *Xenopus oocytes* in 1983 [9, 10]. It is a kind of protein that has the largest distribution among eukaryotes, with a finger domain composed of several cysteine (Cys) and/or histidine (His) and a zinc ion core. Zinc finger protein implicates in regulation of gene expression, cell differentiation and embryo development [11]. MYC associated zinc finger protein (MAZ) is located at 16p11.2 and encodes a 2.7 kb mRNA that translating a molecular weight of approximately 60 KD protein. Studies show that MAZ plays an important role in gene transcription, such as inducing expression of c-Myc, Ras, vascular endothelial growth factor (VEGF) and Podoplanin (PDPN) [12–16], terminating transcription of p53, Sp4 and endothelial nitric oxide synthase (eNOS) [17, 18]. Recent researches indicate deregulated expression of MAZ is closely related to the progression of various tumors, such as glioblastoma, breast cancer, prostate cancer and liposarcoma [14, 19–21]. However, the expression and role of MAZ in HCC has not been explored.

Invasion and metastasis are important malignant manifestation of HCC. Elevated evidences suggested that epithelial-mesenchymal transition (EMT) is a key process of tumor progression, and thought to be an early sign of invasion and metastasis of tumors [8, 22, 23]. The main feature of EMT is cell adhesion molecules (such as E-cadherin) decreased and cytoskeletal proteins (such as Vimentin) increased, resulting in mesenchymal cell like morphology. Epithelial cells lose cell polarity and decrease the connection with the basement membrane through EMT, that is why epithelial cell derived malignant tumor cells acquire ability of invasion and migration [24, 25]. In view of the effect of EMT in HCC invasion and metastasis, it is worth to find out whether MAZ is involved in EMT process of HCC.

In this study, we analyzed the expression of MAZ in tissue samples from HCC patients of Guangxi and explored that MAZ played a role in the pathogenesis and prognosis of HCC. Moreover, we found a close relationship between MAZ and EMT markers by RNA interruption. Our results support MAZ can be a potential biomarker of diagnosis and prognosis of HCC.

## RESULTS

### MAZ is highly expressed in HCC tissues and correlated with distant metastasis of HCC

**Figure 1:**
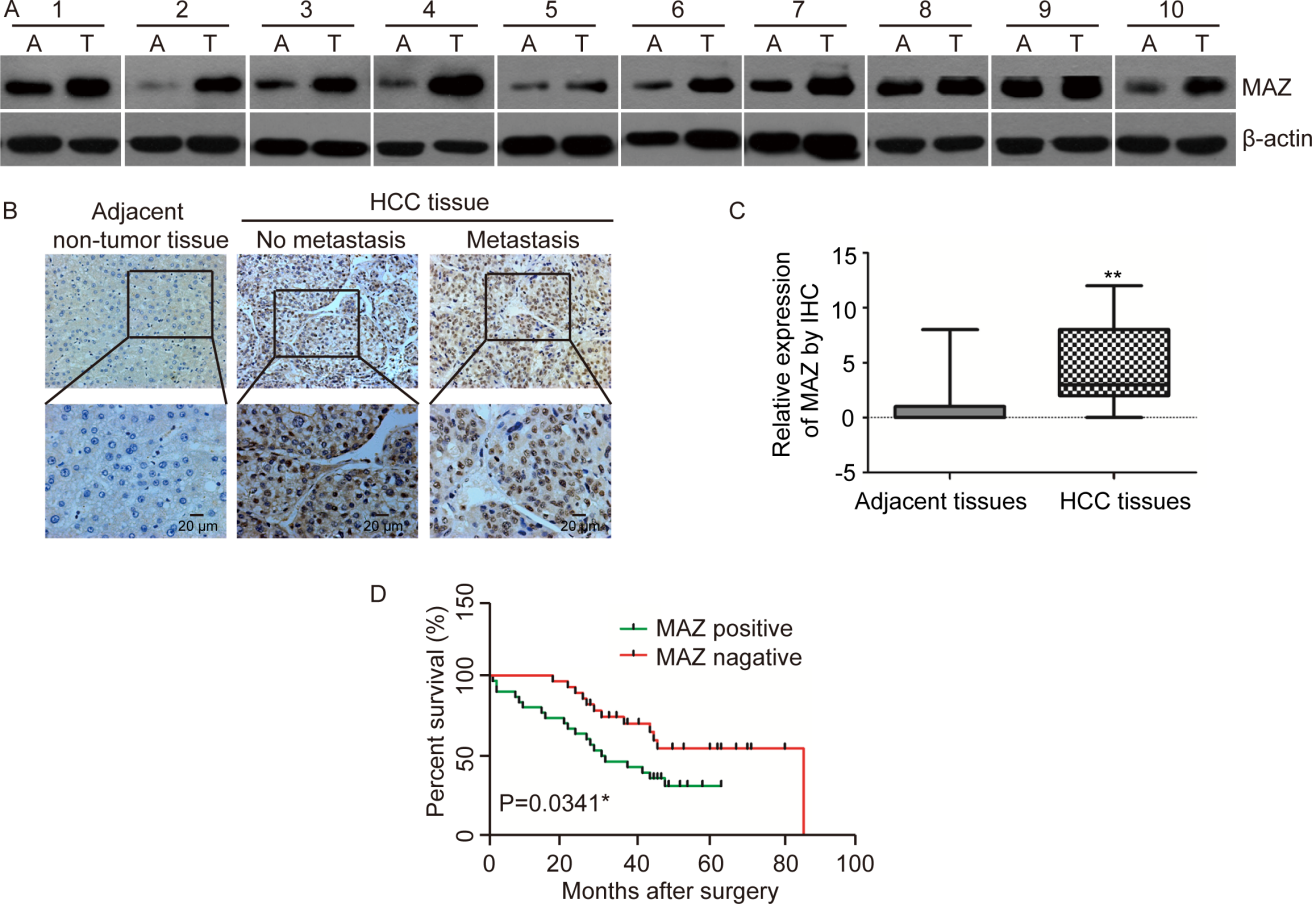
MAZ is highly expressed in HCC and correlated with prognosis of HCC patients (**A**) MAZ protein expression was analyzed in representative HCC (T) and adjacent non-tumor (A) tissues by Western blot. (**B**) MAZ protein expression was detected in representative HCC and adjacent non-tumor tissues by immunohistochemical analysis. No metastasis, HCC without distant metastasis; Metastasis, HCC with distant metastasis. (**C**) statistical analysis of MAZ expression was performed in 75 pairs of HCC and adjacent non-tumor tissues. **P < 0.01 is based on the χ^2^ test. (**D**) correlation of MAZ expression with survival time of HCC patients was conducted by Kaplan-Meier survival analysis.

In order to investigate the relationship between MAZ and HCC, we firstly examined protein expression of MAZ in 23 pairs of HCC and adjacent non-tumor tissues by Western blot. Compared with adjacent non-tumor tissues, MAZ was significantly more highly expressed in 86.9% (20/23) of HCC tissues (Figure 1A). For further prove the expression of MAZ in HCC, we performed immunohistochemical analysis (IHC) to detect MAZ protein in another 75 pairs of HCC and adjacent non-tumor tissue samples. We scored the IHC staining cells and MAZ expression was defined as positive or negative according to the scores mentioned in materials and methods. In accordance with the result from Western blot, expression of MAZ was significantly higher in HCC tissues than in adjacent non-tumor tissues (Figure 1B and 1C). Moreover, when we analyzed MAZ expression in HCC tissues without or with distant metastasis, we found that MAZ over-expression was significantly correlated with distant metastasis of HCC tissues (Figure 1B). Therefore, we collected clinical pathological information of the 75 cases of HCC patients and analyzed the correlation with MAZ expression. As shown in Table 1, there was a significant correlation between MAZ expression with smoking, alcohol intake, tumor diameter and metastasis (*P* < 0.05), while there was no statistical correlation between MAZ with other clinicopathologic parameters of HCC patients, such as sex, age and HBV infection (*P* > 0.05). These results show that MAZ is involved in HCC progression and has a relationship with metastasis of HCC.

**Table 1:** MAZ staining and clinicopathologic characteristics of hepatocellular carcinoma patients

Variables	MAZ staining		χ2 value	P value
	Positive	Negative		
**Sex**				
Male	30	31	0.182	0.669
Female	6	8		
**Age (year)**				
≥ 50	18	23	0.608	0.435
< 50	18	16		
**Smoking**				
Yes	20	12	6.275	**0.012**
No	10	22		
**Alcohol intake**				
Yes	21	15	4.338	**0.037**
No	9	19		
**HCC family history**				
Yes	2	5	0.922	0.337
No	23	25		
**HBV infection**				
Yes	29	29	0.605	0.437
No	2	4		
**Liver cirrhosis**				
Yes	16	12	1.496	0.221
No	20	27		
**Tumor diameter (cm)**				
≥ 5	25	24	4.032	**0.045**
< 5	7	19		
**AFP (ng/ml)**				
≥ 400	17	15	1.233	0.267
< 400	13	20		
**TNM stage**				
I + II	20	22	0.006	0.941
III + IV	16	17		
**Metastasis**				
Yes	28	15	11.829	**0.001**
No	8	24		

Bold values indicate significance. *P* value is based on the χ^2^ test.

To explore the clinical significance of high expression of MAZ in HCC, we conducted Kaplan-Meier survival analysis to compare the survival time of the 75 HCC patients after surgery (Figure 1D). As a result, the survival time of MAZ positive expression group was significantly lower than that of MAZ negative expression group (*P* < 0.05), indicating that high expression of MAZ is correlated with poor prognosis of HCC patients. Collectively, all these results suggest that MAZ plays an important role in HCC progression and prognosis.

### MAZ expression is positively correlated with expression of ZEB1 and ZEB2 in HCC

**Figure 2:**
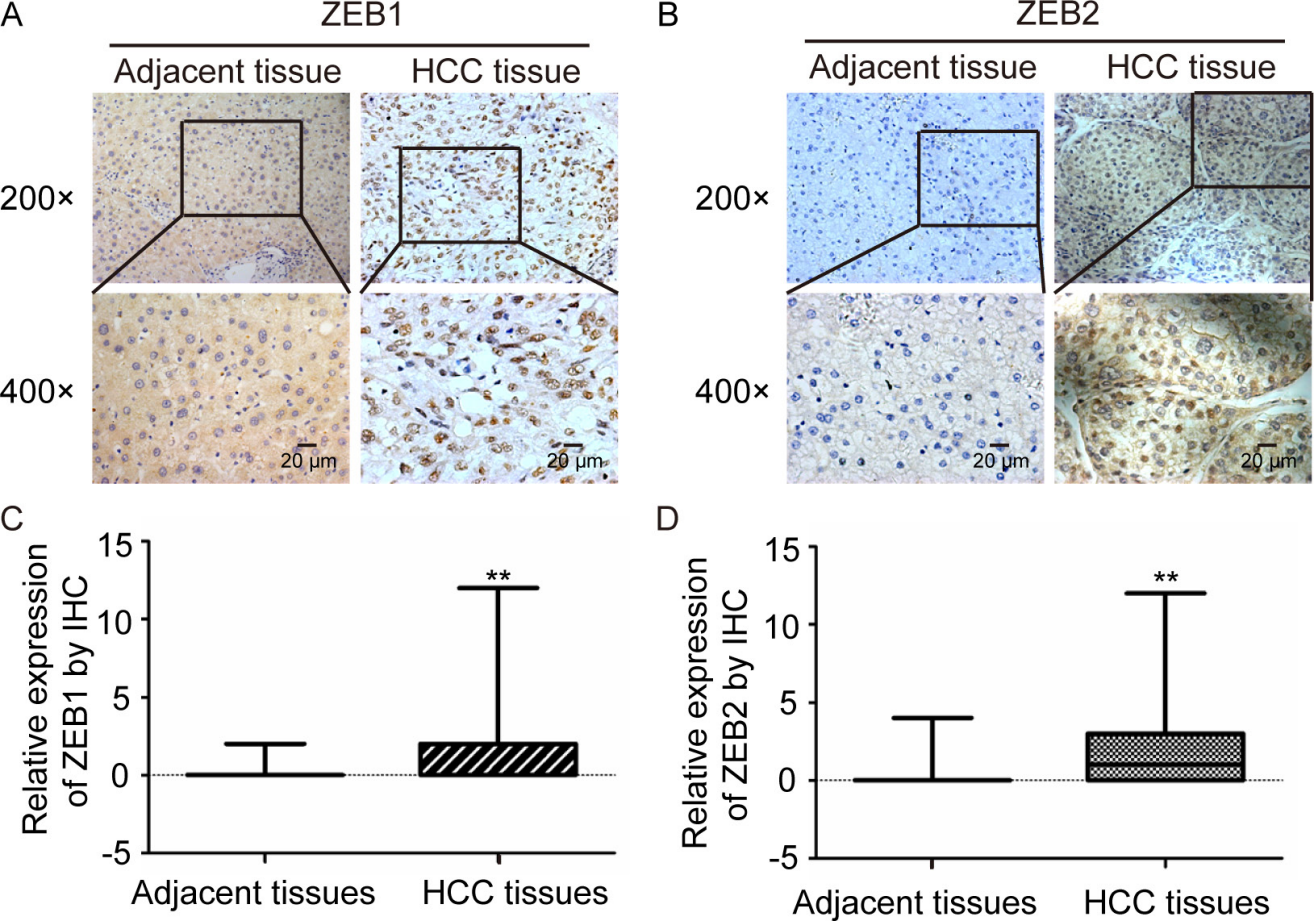
ZEB1 and ZEB2 are over-expressed in HCC tissues (**A** and **B**) ZEB1 and ZEB2 protein expression was detected in representative HCC and adjacent non-tumor tissues by immunohistochemical analysis. (**C** and **D**) statistical analysis of ZEB1 and ZEB2 expression was performed in 75 pairs of HCC and adjacent non-tumor tissues. **P < 0.01 is based on the χ^2^ test.

ZEB1 and ZEB2, has been demonstrated implicated in many cancers as EMT Key factors. By inhibiting E-cadherin expression, ZEB1 was found to cause intercellular adhesion damage and weaken links with the basement membrane, resulting in invasion and metastasis of tumor cells from the primary place [26]. We detected the expression of ZEB1 and ZEB2 in the 75 pairs of HCC and adjacent non-tumor tissues that MAZ expression had been measured by IHC. Expression of ZEB1 and ZEB2 in HCC and adjacent non-tumor tissues was shown in Figure 2A and 2B. Moreover, ZEB1 and ZEB2 were significantly higher expressed in HCC tissues than adjacent non-tumor tissues (Figure 2C and 2D).

As MAZ was also highly expressed in these HCC tissues, we want to find out whether there is a relationship between MAZ with ZEB1 and ZEB2. As shown in Tables 2 and 3, after Spearman correlation analysis, expression of MAZ has a significant positive relationship with ZEB1 (r = 0.548, *P* < 0.05) as well as ZEB2 (r = 0.446, *P* < 0.05). These results imply that MAZ might be involved in EMT process, which promotes the invasion and metastasis of HCC.

**Table 2:** The correlation between MAZ expression and ZEB1 expression in HCC tissues

MAZ expression	ZEB1 expression		χ^2^ value	r value	*p* value
	Positive	Negative			
**Positive**	25	11	22.561	0.548	**0.000**
**Negative**	6	33			

Bold values indicate significance. *P* value is based on Spearman correlation test.

**Table 3:** The correlation between MAZ expression and ZEB2 expression in HCC tissues

MAZ expression	ZEB2 expression		χ^2^ value	r value	*p* value
	Positive	Negative			
**Positive**	28	8	14.921	0.446	**0.000**
**Negative**	13	26			

Bold values indicate significance. *P* value is based on Spearman correlation test.

### MAZ is over-expressed and promotes the proliferation of HCC cells

**Figure 3:**
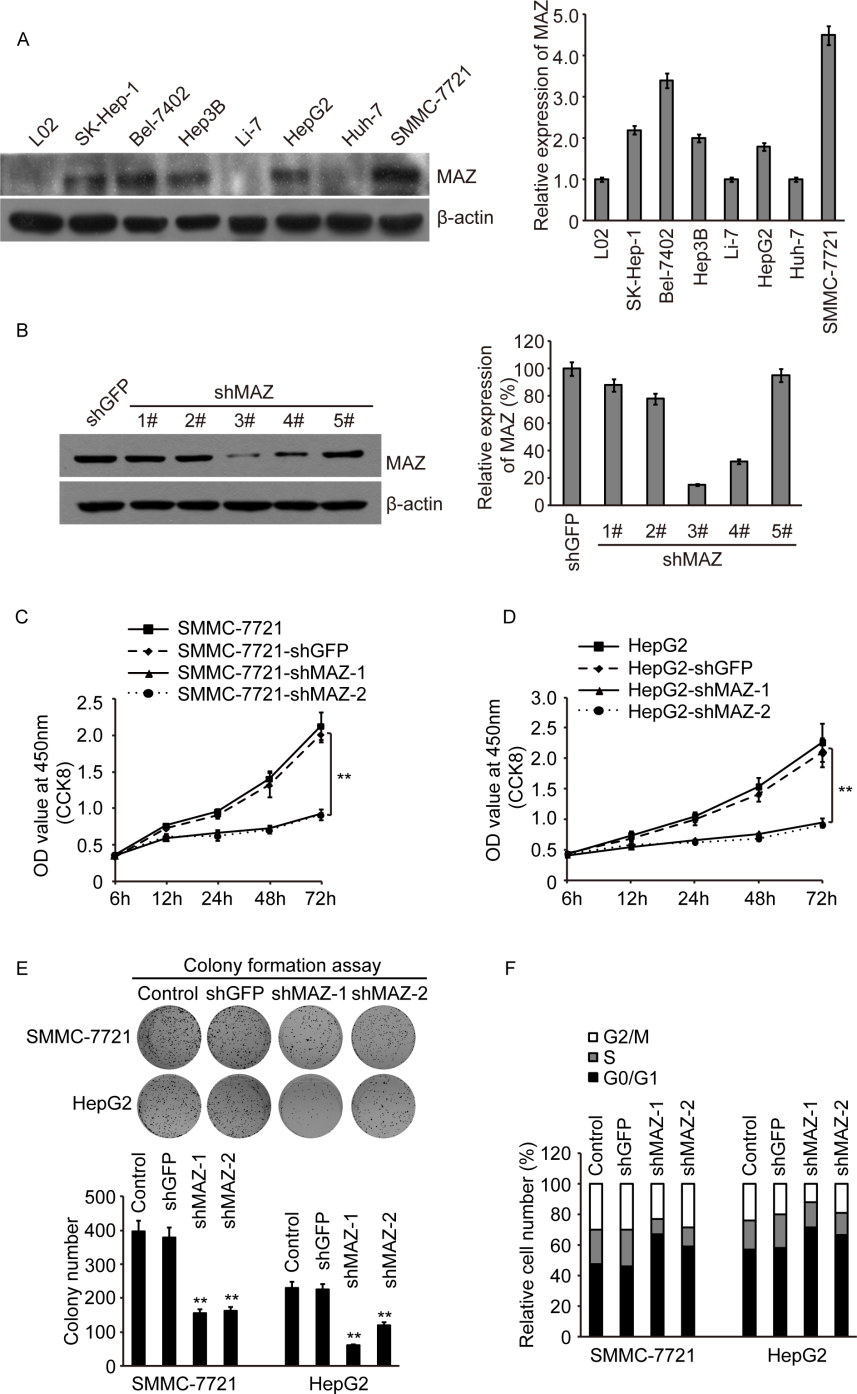
MAZ is over-expressed and promotes the proliferation of HCC cells (**A**) MAZ protein expression in L02 cell line and HCC cell lines as indicated was detected by Western blot (right panel, gray scan results). (**B**) MAZ protein expression in SMMC-7721 cells introduced with five specific shRNAs targeted MAZ and a control shGFP by Western blot (right panel, gray scan results). (**C** and **D**) cell proliferation was detected by CCK-8 in SMMC-7721 and HepG2 cells. **P < 0.01 is based on the Student *t* test compared to the control shGFP cells. (**E**) cell proliferation was detected by colony formation assay in SMMC-7721 and HepG2 cells. Upper panel was representative colony pictures and lower panel was colony number. **P < 0.01 is based on the Student *t* test compared to the control shGFP cells. (**F**) cell cycle profiles of SMMC-7721 and HepG2 cells were determined by flow cytometry.

In order to further clarify the biologic function of MAZ in HCC cells, we firstly detected expression of MAZ in several HCC cell lines, SK-Hep-1, Bel-7402, Hep3B, Li-7, HepG2, Huh-7 and SMMC-7721. Compared with normal liver cell line L02, MAZ was significantly increased in these HCC cell lines, especially in Bel-7402 and SMMC-7721 cells (Figure 3A). At the same time, we purchased 5 shRNAs targeting MAZ (shMAZ) and a control plasmid shGFP. After introducing these shRNAs to SMMC-7721 cell line, we found out No.3 and No.4 of shMAZ could knock down more than 70% of MAZ protein (Figure 3B). Finally, we selected two cell lines (SMMC-7721 and HepG2) and two shMAZs (No.3 and No.4) to construct MAZ knock-down cells for the following study.

After MAZ knock-down cell lines were established, we detected the cell proliferation firstly by CCK-8 assay. We found that knocking-down of MAZ significantly decreased the proliferation rate of SMMC-7721 and HepG2 cells (Figure 3C and 3D). The same result was also proved in colony formation assay (Figure 3E). In addition, as MAZ was reported to suppress the transcription of p53 [27], we detected the cell cycle profile of MAZ knock-down cells. Compared with the control shGFP cells, G0/G1 phase of shMAZ cells were increased by 10%–20% (Figure 3F), suggesting MAZ knock-down could cause a G0/G1 phase arrest. These results indicate that MAZ is highly expressed in HCC cells and promotes the proliferation of HCC cells.

### MAZ promotes tumorigenesis *in vivo*

**Figure 4:**
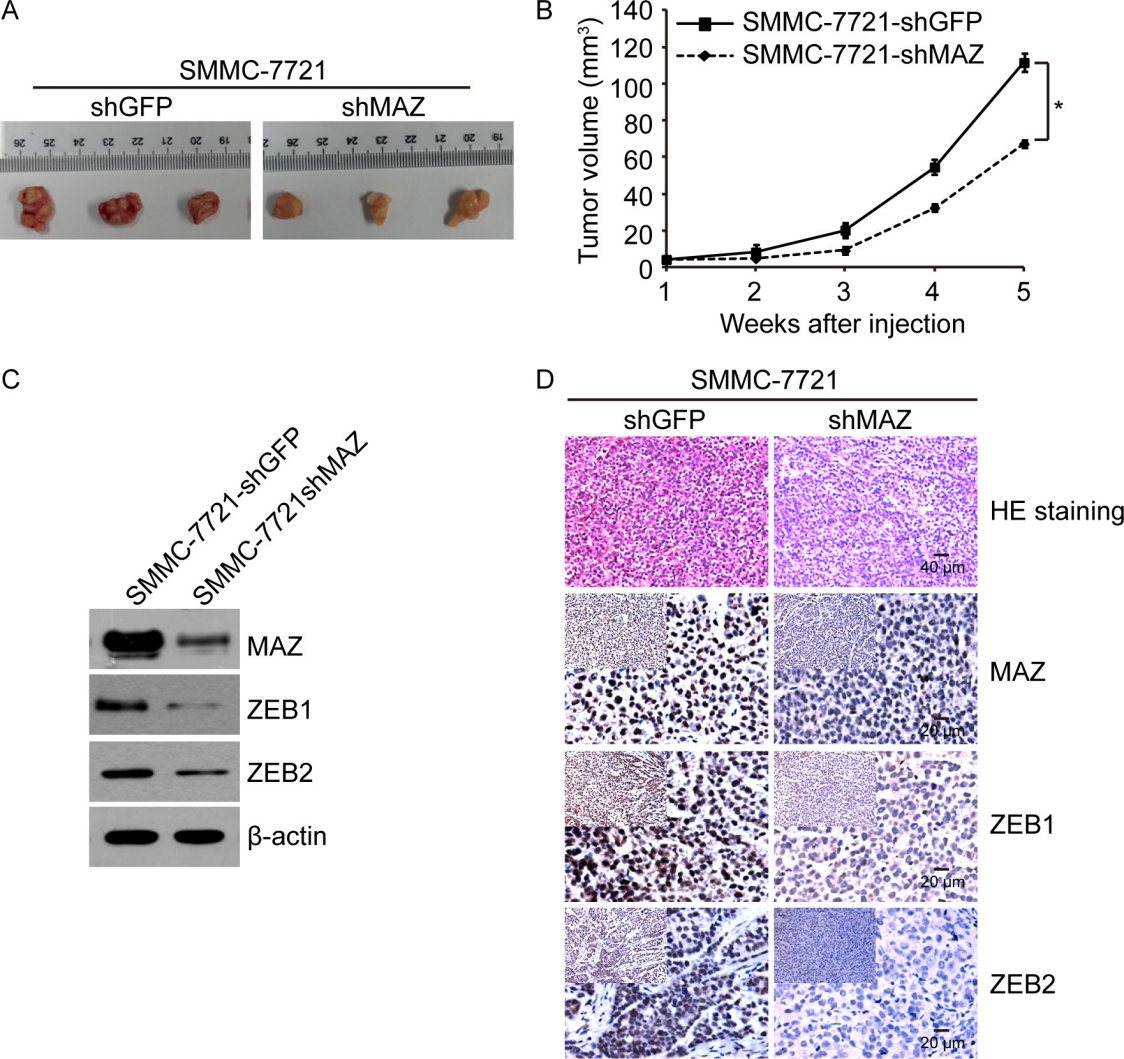
MAZ promotes tumorigenesis *in vivo*. (**A**) representative images of SMMC-7721-shMAZ and the control SMMC-7721-shGFP cell tumors by subcutaneous injection. (**B**) volume growth of tumors formed by SMMC-7721-shMAZ and the control SMMC-7721-shGFP cells in nude mice. *P < 0.05 is based on the Student *t* test compared to SMMC-7721-shGFP cells. (**C**) protein expression of SMMC-7721-shMAZ and the control SMMC-7721-shGFP cell tumors in nude mice by Western blot. (**D**) protein expression of SMMC-7721-shMAZ and the control SMMC-7721-shGFP cell tumors in nude mice by immunohistochemical analysis.

To extend our *in vitro* observations, we investigated whether MAZ could regulate tumorigenic of HCC cells *in vivo*. 20 healthy nude mice were randomly divided into two groups. One group of nude mice was subcutaneously injected with SMMC-7721-shMAZ cells and the other group was subcutaneously injected with the control SMMC-7721-shGFP cells. Tumor size of nude mice was measured every week up to 5 weeks. In accordance with the cell result *in vitro*, knocking-down of MAZ expression in SMMC-7721 cells led to a significant decrease in volume of tumors formed in nude mice after 5 weeks (Figure 4A). Moreover, tumor volume of SMMC-7721-shMAZ cells grew slower at the implantation site than the control SMMC-7721-shGFP cells (Figure 4B). As there was a positive correlation between MAZ with ZEB1 and ZEB2, we detected the expression of ZEB1 and ZEB2 in nude mice tumors by Western blot and IHC (Figure 4C and 4D). ZEB1 and ZEB2 decreased when the expression of MAZ was knocked-down, further supporting the results in HCC tissues. These *in vivo* results further demonstrate the critical role of MAZ in HCC tumorigenesis.

### MAZ induces EMT by regulating expression of EMT markers

**Figure 5:**
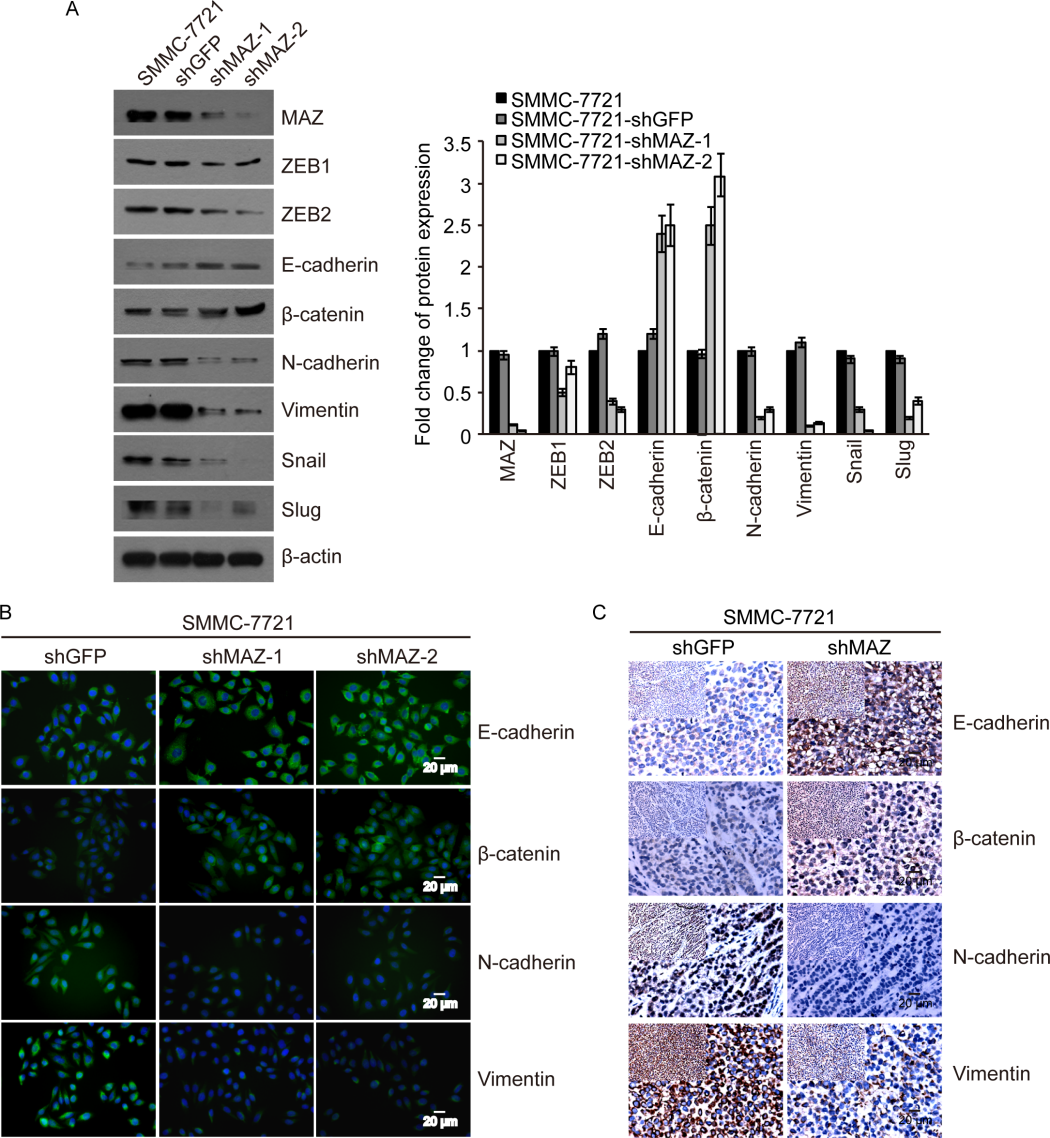
MAZ induces EMT by regulating expression of EMT markers (**A** and **B**) protein expression of EMT markers indicated was detected in SMMC-7721 cells by Western blot and immunofluorescence. (**C**) protein expression of EMT markers indicated was detected in nude mice tumors formed from SMMC-7721 cells by immunohistochemical analysis. Pictures were photographed under 400× magnification and pictures of top left panel were photographed under 200× magnification.

Because ZEB1 and ZEB2 are key EMT factors, we want to find out whether MAZ can induce EMT to promote HCC cell migration and invasion by regulating EMT process. We purchased an EMT antibody kit to detect the expression of EMT markers including ZEB1 and ZEB2 in MAZ knock-down cells. As shown in Figure 5A, compared to the control SMMC-7721-shGFP cells, epithelial markers (E-cadherin and β-catenin) were increased by 120%–200%, while mesenchymal markers (N-cadherin, Vimentin, Snail, Slug, ZEB1 and ZEB2) were decreased by 20%–90% in SMMC-7721-shMAZ cells. This result was also confirmed by immunofluorescence analysis that expression of EMT changed by reducing MAZ protein (Figure 5B). And expression of EMT markers in HepG2 cells detected by Western blot and immunofluorescence was the same as that in SMMC-7721 cells (Supplementary Figure S1A and S1B). Moreover, E-cadherin and β-catenin were increased, while N-cadherin and Vimentin were decreased in tumors formed by SMMC-7721-shMAZ cells (Figure 5C). These results show that MAZ can regulate expression of EMT markers to induce HCC invasion and metastasis.

### MAZ promotes invasion and metastasis of HCC cells

**Figure 6:**
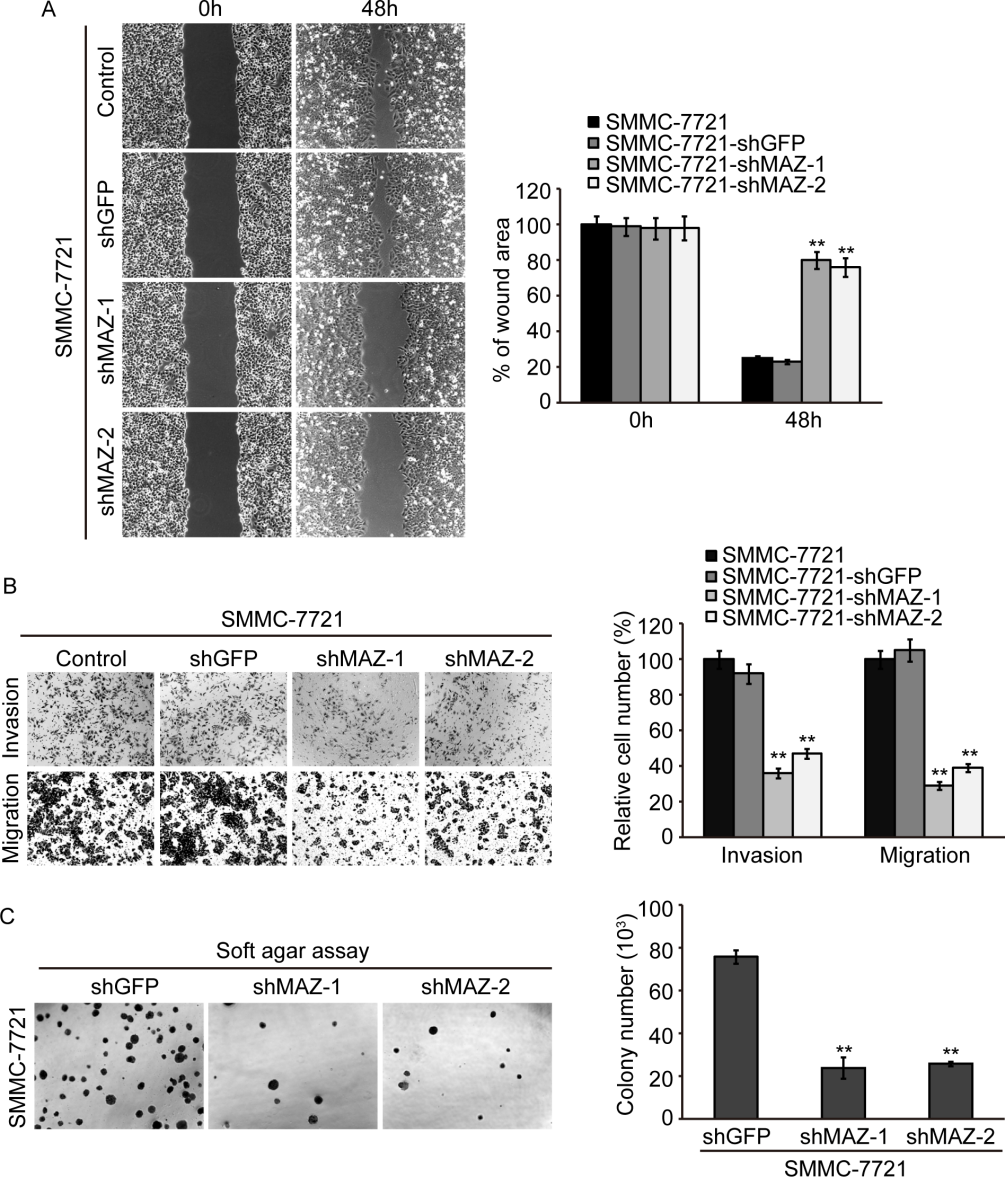
MAZ promotes invasion and metastasis of HCC cells (**A** and **B**) invasion and migration ability of cells was analyzed by wound healing and Transwell assay. (**C**) representative colony pictures and colony number count in SMMC-7721 cells by soft agar assay. **P < 0.01 is based on the Student *t* test compared to SMMC-7721-shGFP cells. All results are from three independent experiments.

To confirm the role of MAZ plays in invasion and metastasis of HCC, we measured the invasion and migration ability of SMMC-7721 cells after decreasing MAZ expression. Wound healing assay was performed to detect tumor cell migration. As shown in Figure 6A, wound area of SMMC-7721-shMAZ cells was significantly larger than control SMMC-7721-shGFP cells at 48 h after wound time (*P* < 0.05). This result was also confirmed by Transwell assay (Figure 6B). Both invasion and migration were significantly decreased when MAZ expression was knocked-down in SMMC-7721 cells (*P* < 0.05). In addition, the size of colonies formed in soft agar assay by SMMC-7721-shMAZ cells was smaller, and the number of colonies was decreased as compared with the colonies formed by SMMC-7721-shGFP cells (Figure 6C). The results of wound healing, Transwell and soft agar assay in HepG2 cells were the same as that of SMMC-7721 cells (Supplementary Figure S2A, S2B and S2C). These results support that MAZ promotes invasion and migration of HCC cells by targeting EMT process.

## DISCUSSION

HCC, a common malignant tumor, is one of the main death causes around the world, and ranks third in cancer death spectrum of China [2]. The high mortality of HCC results from obscure occurrence, high malignancy, late diagnosis, high recurrence and metastasis after surgery [28]. However, recent clinical therapies to HCC such as surgery, hepatic arterial infusion chemotherapy, transcatheta arterial chemical embolism (TACE), radiotherapy and molecular targeted therapy are not satisfactory [4, 28, 29]. There is still a great need of good diagnostic markers, drug targets and therapeutic strategies for successful treatment of HCC.

Carcinogenesis is a multistep process that many genes involved, such as activation of proto-oncogenes and inactivation of tumor suppressor genes [1]. Zinc finger protein plays an important role in regulation of gene expression, cell differentiation and carcinogenesis [11]. MAZ, as a member of zinc finger protein, not only plays an important role in the regulation of blood-brain barrier and colitis caused by hypoxia [30, 31], but also regulates gene expression of p53, c-Myc, VEGF, Ras, PDPN and caveolin-1 [13–16, 27, 30]. Moreover, MAZ implicates in carcinogenesis and development of many tumors. Previous report showed over-expression of MAZ in breast cancer and affected the prognosis of patients with breast cancer by up-regulating miR-34a [32]. MAZ was also increased in prostate cancer cells and positively transcriptional regulated androgen receptor. When MAZ was knocked-down, cell proliferation, invasion and migration ability of prostate cancer cells were decreased [20]. In addition, MAZ promoted tumor angiogenesis in human glioblastoma through transcriptional regulation of VEGF [15] and controlled liposarcoma cell proliferation and apoptosis through directly regulating GNDF in RET signaling with synergy interaction of SPN1 [19]. However, its role and underlying mechanisms in HCC is still unknown.

Our study show an oncogene role of MAZ plays in HCC for the first time. We found that MAZ was over-expressed in HCC tissues, and correlated with tumor diameter and distant metastasis. Survival time of HCC patients with high MAZ expression was significantly decreased than HCC patients with low MAZ expression. Moreover, we knocked-down expression of MAZ in HCC cells by shRNA to prove the role of MAZ in HCC. We found out that cell proliferation of shMAZ was significantly inhibited by CCK-8 and colony formation assay. The same result was also demonstrated *in vivo*. These results suggest that MAZ plays an important role in HCC tumorigenesis and prognosis.

Invasion and metastasis are essential biological characteristics of malignant tumors. EMT, as one form of tumor invasion and metastasis, is a process necessary for many malignant tumors [22, 33]. ZEB1 and ZEB2, as key factors of EMT, have been confirmed to inhibit expression of E-cadherin [26, 34–36] to promote invasion and metastasis of tumors by inducing EMT [23, 37–40]. Over-expression of ZEB1 was found in HCC with the inhibition of E-cadherin and correlated with poor prognosis of HCC patients [41]. ZEB2 was also reported to promote invasion and metastasis of gastric cancer by inhibiting E-cadherin [42]. In order to clarify the relationship between MAZ, ZEB1 and ZEB2 in HCC, we detected their expression in 75 pairs of HCC and adjacent non-tumor tissues. We found that the expression of ZEB1 and ZEB2 was positively correlated with MAZ. After knocking-down MAZ expression, expression of epithelial markers (E-cadherin and β-catenin) was increased and expression of mesenchymal markers (N-cadherin, Vimentin, Snail, Slug, ZEB1 and ZEB2) was reduced by Western blot and immunofluorescence detection. In addition, the invasion and migration ability of MAZ knock-down cells was significantly decreased through Transwell, wound healing test and soft agar assay. These results suggest that MAZ contributes to the invasion and metastasis of HCC by inducing EMT.

Collectively, our results show a critical role of MAZ plays in HCC and elaborate an EMT mechanism MAZ might be involved. Our study provides a basis for MAZ as a potential target in screening of HCC susceptible population, therapeutic intervention and clinical prognosis.

## MATERIALS AND METHODS

### Reagents and antibodies

Lipofecatmine 2000 for cell transfection was purchased from Thermo Fisher Scientific (Waltham, MA, USA). Antibodies against MAZ, ZEB1 and ZEB2 were purchased from Abcam (Cambridge, MA, USA). Epithelial-Mesenchymal Transition (EMT) Antibody Sampler Kit was from Cell Signaling technology (Danvers, MA, USA). Anti-β-actin antibody was from Signalway Antibody LLC (College Park, Maryland, USA). Unless otherwise noted, all other reagents were from Sigma-Aldrich (St. Louis, MO, USA).

### Patient and tissue samples

23 cases of hepatocellular carcinoma (HCC) and paired adjacent non-tumor tissues for Western blot were collected from HCC patients under surgery in the Affiliated Hospital of Guilin Medical University between 2014 and 2015. All samples were frozen in liquid nitrogen. Another 75 cases of HCC and paired adjacent non-tumor tissues for immunohistochemical detection were obtained from Department of Pathology, the Affiliated Hospital of Guilin Medical University during 2005–2009. Tumor staging of the 75 HCC tissues was based on the 6th edition of the tumor-node-metastasis (TNM) classification of the International Union against Cancer and the clinicopathologic characteristics were summarized in Table 1. Written informed consent was obtained from patients and approved by the Hospital Ethics Committee of Guilin Medical University.

### Immunohistochemical analysis and scoring

75 paired paraffin-embedded tissues were made into tissue microarray by Fanpu (Guilin, China). The tissue microarray was first dewaxed by dimethylbenzene and hydrated by ethanol. After incubated in 0.1% EDTA for 2 min for antigen repair by high pressure heating, the tissue microarray was soaked in 3% hydrogen peroxide for 10 min to remove endogenous peroxidase. Then it was blocked for 10 min by sheep serum and incubated with primary antibodies for 1 h at 37°C. After 30 min of incubation with secondary antibodies, the tissue microarray was color-developed by DAB and observed under microscope for 5∼10 min until the appropriate color appeared. And then it was counterstained with hematoxylin, dehydrated, transparent and fixed. All of the immunostained sections were evaluated blindly without any knowledge of the clinic-pathological material information. For assessment, five fields in each specimen were selected randomly. The IHC staining intensities were scored into 4 grades by the brown color in cell nucleus: 0 for no, 1 for light, 2 for middle and 3 for dark brown color. More than 500 cells were counted to determine the mean percentage of immunostained cells relative to the total number of cells. Positive cell staining percentages were scored into four categories: 0 for ≤ 5%, 1 for 6–25%, 2 for 26–50%, 3 for 51–75% and 4 for > 75% staining. The sum of the percentage and intensity scores was used as the final MAZ, ZEB1 and ZEB2 staining score. The staining scores were defined as negative expression for scores < 4 and positive expression for scores ≥ 4.

### Cell lines and plasmids

SK-Hep-1, HepG2, Hep3B, SMMC-7721 and Huh-7 cells were purchased from ATCC (Manassas, VA, USA) and cultured in Dulbecco's modified Eagle medium with 10% fetal bovine serum (FBS). Bel-7402, Li-7 and L02 cells were cultured in RPMI-1640 medium with 10% FBS. All the cell lines were grown at 37°C in a 5% CO_2_/95% air atmosphere.

5 shRNAs that targeted MAZ and a shRNA control were constructed in a lentiviral vector and purchased from Genechem (Shanghai, China). The shRNAs were transferred to SMMC-7721 cells and we chose the 3# and 4# of shMAZ (target sequence: GCCCTTCAAATGTGAGAAA and GGCCATGTTCCCGGTGTTT) with best knock-down effect for the follow-up analyses.

### Western blot

Cells or tissues were lysed with RIPA buffer and the concentration of protein was determined by BCA. 30 μg of protein per hole was loaded for SDS-PAGE electrophoresis. After electrophoresis, the proteins were transferred to PVDF membrane and coated with specific primary antibodies overnight at 4°C after blocking by 5% fat free milk. Before incubation with secondary antibodies at room temperature for 1 h, the membranes were cleaned by TBST buffer. Finally, the bands were visualized by chemoluminescence.

### Cell proliferation test

Cell proliferation was detected using Cell Counting Kit (CCK8, Beyotime, Shanghai, China) according to the manufacturer's protocol. Briefly, cells were grown in triplicate in a 96-well plate with a cell density of 10^4^/well. OD value at 450 nm was detected on a microplate reader at 6 h, 12 h, 24 h, 48 h and 72 h, respectively.

### Colony formation assay

Cells were grown in triplicate in a 6-well plate with a cell density of 800/well and cultured for 2 weeks. After washed twice by PBS, cells were fixed by 4% poly formaldehyde for 15 min and stained by crystal violet for a night. Cells were washed 3 times by de-ionized water and then natural dried at the next day. Cell colony was photographed and counted under microscope.

### Cell cycle detection

Cells were harvested after trypsinized, and washed twice with ice-cold PBS. Then, cells were fixed with 75% cold ethanol overnight at −20°C. After washing twice with ice-cold PBS, fixed cells were centrifuged at 1200 rpm for 5 min and resuspended in 400 μl ice-cold PBS, and then incubated with RNase (Sigma) in a 37°C water-bath for 30 min. Finally, cells were subjected to flow cytometric analyses after 30 min incubation with propidium iodide (PI) at 4°C in the dark.

### Subcutaneous tumor formation test in nude mice

Nude mice and related feeding goods were purchased from the SLAC Laboratory Animal Company (Changsha, China). All animals were used in accordance with institutional guidelines and the current experiments were approved by the Use Committee for Animal Care. 5 × 10^6^ of SMMC-7721 cells were resuspended in 200 μl PBS and inoculated subcutaneously into the 4-week-old nude mice. The tumors were measured weekly and the tumor volume was calculated according to the formula 3.14 × length × width^2^/6. Five weeks later, the tumors were removed from the nude mice and photographed. And then, the tumors were frozen at −80°C for Western blot, or fixed in 10% paraformaldehyde for immunohistochemical analysis.

### Immunofluorescence

After seeded in a 24-well plate for 24 h, cells were cleaned by PBS buffer and fixed with 4% formaldehyde. Then cells were treated with 0.1%Triton X-100 and blocked in 5% FBS at room temperature. After incubated with primary antibodies for a night at 4°C and secondary Goat Anti-Rabbit IgG H&L (Alexa Fluor^®^ 488, Abcam) antibodies for 1 h at 37°C, cells were washed with PBS for three times and stained with DAPI for 5 min. Finally, the cells were observed and photographed under a fluorescence microscope.

### Wound healing assay

Cells were seeded in 6-well plates and wounded by scratching with sterile plastic 10 μl micropipette tips until the cells just covered the plate at a monolayer. Then cells were cleaned and added fresh serum free medium. The cells were photographed 0 h, 24 h and 48 h after the wounding by the phase contrast microscope. Cell migration distance was observed in the photographs.

### Cell ability of invasion and migration analysis

Cell invasion was detected using Matrigel (BD, Franklin lakes, NJ, USA) coated BD Transwell chamber and cell migration was detected using BD Transwell chamber without Matrigel coated. The chamber aperture is 8 μm. The chamber was put in a 24-well plate with medium and activated by incubation for 30 min in an incubator. Then 100 μl of 2 × 10^4^ cells in serum free medium was added to the upper chamber and 600 μl medium with 10% FBS was added to the lower chamber. After 30 h of incubation, cells were fixed with 4% poly formaldehyde and stained by 1% crystal violet. The Matrigel and the remained cells were wiped off. After cleaned by PBS, the cells were photographed and counted.

### Soft agar assay

The soft-agar assay was performed according to the method of Macpherson and Montagnier [43] with minor modifications. Briefly, 1 ml lower layer of 1% agar in DMEM containing 10% FBS was placed in a 6-well plate and solidified at room temperature. After that, 1 × 10^5^ cells were suspended in 1 ml upper layer of 0.5% agar in DMEM with 10% FBS. Each cell line was set up in triplicates and incubated in cell incubator for 3 weeks. The colony was examined with an inverted phase microscope. Groups of 50 or more cells were considered as colonies.

### Statistical analysis

All the results were analyzed using SPSS 19.0. The correlation between clinical-pathological parameters and MAZ expression was analyzed using χ^2^ test. The survival probability was estimated by Kaplan-Meier method, and the comparison of survival curves between groups was done with the log rank test. The level of statistical significance was set at *P* < 0.05 for all tests.

## SUPPLEMENTARY MATERIALS FIGURES AND TABLES


