# Supplementary material for: MYC associated zinc finger protein promotes the invasion and metastasis of hepatocellular carcinoma by inducing epithelial mesenchymal transition
Source: Oncotarget. 2016 Nov 16;7(52):86420–32. doi: 10.18632/oncotarget.13416 (PMC5349923; doi:10.18632/oncotarget.13416)
Supplement: Supplementary file 1 [file oncotarget-07-86420-s001.pdf]

MYC associated zinc finger protein promotes the invasion and metastasis of hepatocellular carcinoma by inducing epithelial mesenchymal transition

Supplementary Materials

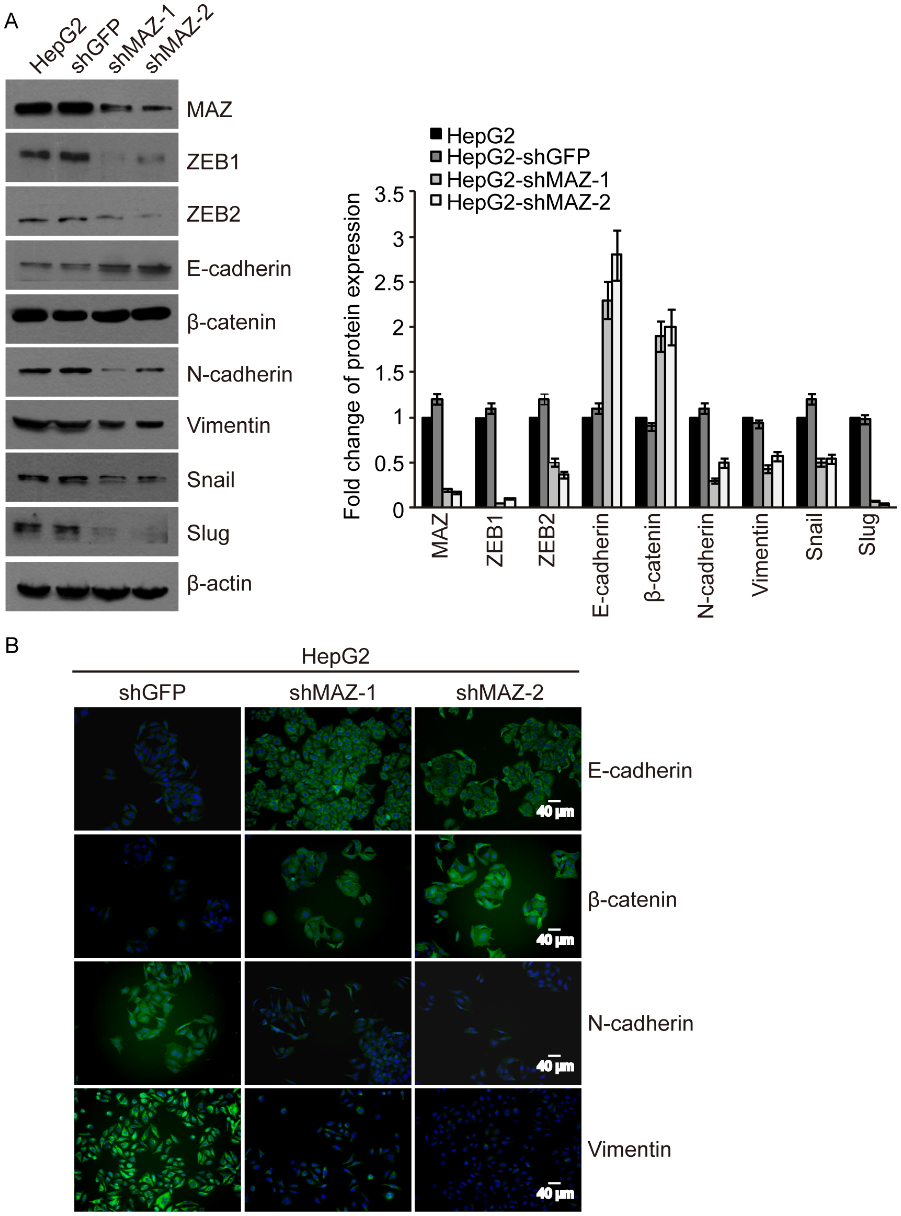

Supplementary Figure S1: (**A** and **B**) protein expression of EMT markers indicated was detected in HepG2 cells by Western blot and immunofluorescence (200× magnification).

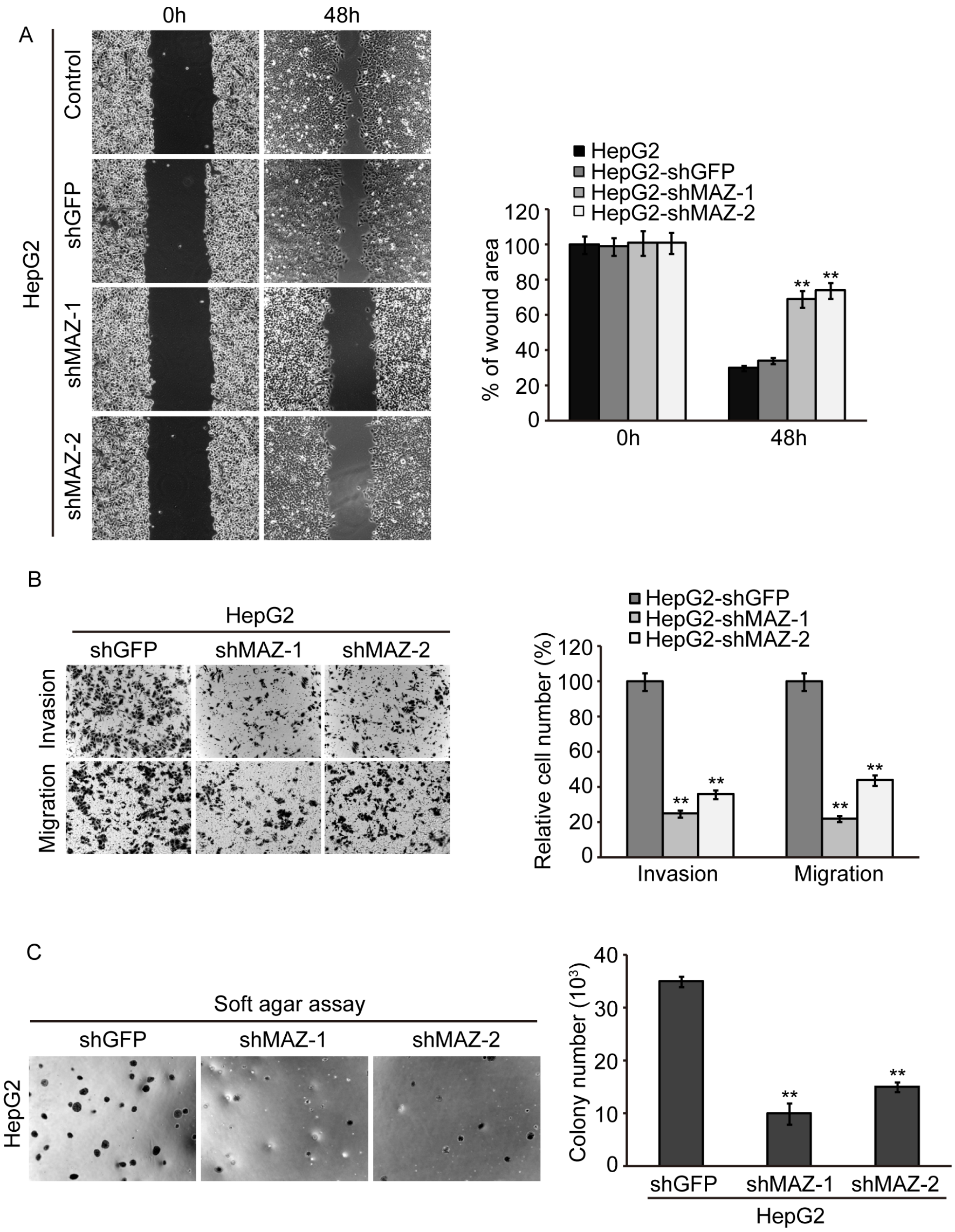

Supplementary Figure S2: (**A** and **B**) invasion and migration ability was analyzed by wound healing and Transwell assay in HepG2 cells. (**C**) representative colony pictures and colony number count in HepG2 cells by soft agar assay. $^{**}P < 0.01$ is based on the Student *t* test compared to HepG2-shGFP cells. All results are from three independent experiments.
